# Population food intake clusters and cardiovascular disease incidence: a Bayesian quantifying of a prospective population-based cohort study in a low and middle-income country

**DOI:** 10.3389/fnut.2023.1150481

**Published:** 2023-07-13

**Authors:** Reyhaneh Rikhtehgaran, Khadijeh Shamsi, Elnaz Mojoudi Renani, Arman Arab, Fatemeh Nouri, Noushin Mohammadifard, Hamid Reza Marateb, Marjan Mansourian, Nizal Sarrafzadegan

**Affiliations:** ^1^Department of Mathematical Sciences, Isfahan University of Technology, Isfahan, Iran; ^2^Student Research Committee, Department of Epidemiology and Biostatistics, School of Health, Isfahan University of Medical Sciences, Isfahan, Iran; ^3^Department of Community Nutrition, School of Nutrition and Food Sciences, Isfahan University of Medical Sciences, Isfahan, Iran; ^4^Isfahan Cardiovascular Research Center, Cardiovascular Research Institute, Isfahan University of Medical Sciences, Isfahan, Iran; ^5^Hypertension Research Center, Cardiovascular Research Institute, Isfahan University of Medical Sciences, Isfahan, Iran; ^6^Department of Biomedical Engineering, Faculty of Engineering, University of Isfahan, Isfahan, Iran; ^7^Pediatric Cardiovascular Research Center, Cardiovascular Research Institute, Isfahan University of Medical Sciences, Isfahan, Iran

**Keywords:** Bayesian cluster analysis, cardiovascular diseases, food intake patterns, dietary pattem, heart health

## Abstract

**Aims:**

This study was designed to explore the relationship between cardiovascular disease incidence and population clusters, which were established based on daily food intake.

**Methods:**

The current study examined 5,396 Iranian adults (2,627 males and 2,769 females) aged 35 years and older, who participated in a 10-year longitudinal population-based study that began in 2001. The frequency of food group consumption over the preceding year (daily, weekly, or monthly) was assessed using a 49-item qualitative food frequency questionnaire (FFQ) administered *via* a face-to-face interview conducted by an expert dietitian. Participants were clustered based on their dietary intake by applying the semi-parametric Bayesian approach of the Dirichlet Process. In this approach, individuals with the same multivariate distribution based on dietary intake were assigned to the same cluster. The association between the extracted population clusters and the incidence of cardiovascular diseases was examined using Cox proportional hazard models.

**Results:**

In the 10-year follow-up, 741 participants (401 men and 340 women) were diagnosed with cardiovascular diseases. Individuals were categorized into three primary dietary clusters: healthy, unhealthy, and mixed. After adjusting for potential confounders, subjects in the unhealthy cluster exhibited a higher risk for cardiovascular diseases [Hazard Ratio (HR): 2.059; 95% CI: 1.013, 4.184] compared to those in the healthy cluster. In the unadjusted model, individuals in the mixed cluster demonstrated a higher risk for cardiovascular disease than those in the healthy cluster (HR: 1.515; 95% CI: 1.097, 2.092). However, this association was attenuated after adjusting for potential confounders (HR: 1.145; 95% CI: 0.769, 1.706).

**Conclusion:**

The results have shown that individuals within an unhealthy cluster have a risk that is twice as high for the incidence of cardiovascular diseases. However, these associations need to be confirmed through further prospective investigations.

## Introduction

Non-communicable diseases (NCDs) are the leading cause of death worldwide, accounting for 63% of all deaths. Traditionally, NCDs have been perceived as a burden predominantly on developed countries. However, recent evidence suggests a reverse trend and a dramatic increase in NCDs in low- and middle-income countries (LMICs) (1). NCDs primarily include chronic respiratory diseases, cancers, diabetes, and cardiovascular diseases (CVDs), which are the most frequent causes of mortality in most Western Pacific, South-East Asian, European, Eastern Mediterranean, and American countries (2).

Risk factors for NCDs such as harmful alcohol consumption, a sedentary lifestyle, tobacco use, and an unhealthy diet—characterized by high consumption of foods rich in sugar, salt, trans and saturated fats—can increase the risk of complications in individuals with NCDs (3). Among these established risk factors, diet plays a pivotal role, particularly concerning CVDs. Over the past few decades, numerous studies have improved our understanding of the correlation between diet and cardiovascular health (4, 5). The focus of these studies has shifted from investigating individual nutrients and foods to evaluating dietary patterns, reflecting the synergistic and combined effects of various foods and beverages (6). Dietary pattern analysis considers the cumulative effects of the overall diet and the complex interactions between foods and nutrients (7).

A traditional approach to dietary pattern analysis is factor analysis, which empirically derives dietary patterns by reducing the dimensionality of dietary data. In factor analysis, the covariance matrix of food intakes is used to generate linear combinations (factors), and participants are assigned a score based on these derived factors (8). However, since each participant may belong to more than one factor, interpreting these scores can be challenging (9, 10). Another approach is cluster analysis, which, unlike factor analysis, classifies participants into mutually exclusive, non-overlapping clusters based on the similarity of their dietary intake. This means members of each cluster have similar dietary intakes and are different from members of other clusters (9). Cluster analysis has advantages over factor analysis as it can classify individuals into distinct groups, enabling the comparison of nutritional differences among people in different groups and determining whether there is a relationship between dietary pattern subgroups and health outcomes (11).

The majority of current literature exploring the link between dietary patterns and the risk of CVDs employs factor analysis and suggests an inverse relationship between healthy eating patterns and the incidence of CVDs (12–15). However, less evidence is available regarding the use of cluster analysis to examine the relationship between diet and the risk of CVDs (16–18). In addition to the conflicting findings of previous studies, most of them have primarily focused on high-income and industrialized nations (19).

In recent decades, Iran has experienced significant changes in various social and economic aspects (20). As a middle-income country, Iran’s economic growth has been subject to fluctuations, influenced by international sanctions, global oil prices, trade policies, domestic economic policies, and geopolitical tension. These economic changes have prompted many Iranians to modify their dietary patterns due to the need for lower-cost options. Social changes have also significantly influenced this transition. The nutritional transition in Iran has led to a shift toward Western dietary patterns (21).

Given the significant burden of cardiovascular diseases in Iran and the importance of identifying their underlying causes, this cohort study aimed to identify major dietary clusters among the general adult population of Iran using Bayesian cluster analysis. Moreover, the study sought to explore the relationship between these dietary clusters and the incidence of CVDs.

## Method

### Study participants and setting

The present study involved 5,396 individuals who participated in the Isfahan Cohort Study (ICS). The ICS is a population-based, longitudinal study that includes participants who were at least 35 years old at baseline and resided in urban or rural areas of three central counties of Iran, namely Isfahan, Arak, and Najaf-Abad (22). These cities exhibit similar general population characteristics and geographic variables. These participants had previously engaged in the Isfahan Healthy Heart Program (IHHP) baseline survey, which is a community trial for CVD prevention and control (22). The ICS study was executed by the World Health Organization (WHO) collaborating center, the Ethical Committee of the Isfahan Cardiovascular Research Center (ICRC). The primary aim of the ICS study was to determine the individual and combined impacts of different risk factors on the incidence of CVD events, including fatal and nonfatal coronary artery diseases and stroke. Participants have been monitored since 2003, with follow-ups conducted *via* phone calls for cardiovascular events every 2 years and risk factor interviews every 5 years (22). The selection of participants was facilitated through a multistage cluster random sampling method based on the distributions of sex, age, and residential areas (urban/ rural). A detailed protocol for the study has been previously described (22). The study included those who had lived for at least 6 months in Isfahan, Arak, or Najaf-Abad, had Iranian nationality and were mentally competent. Exclusions from the ICS baseline survey were made for participants with a history of myocardial infarction (MI), stroke, heart failure, or pregnancy. After 2001, participants were followed up through biennial phone interviews until 2013 to record cardiovascular outcomes. The phone interviews included inquiries about the participant’s survival, cause of death (if applicable), cardiovascular events, cerebrovascular events, neurological symptoms, and hospitalizations. If any outcomes of interest occurred, participants were asked for further information. This included reviewing relevant health records and questionnaires, and in the case of non-hospital deaths, examining death certificates from the death database, and conducting a verbal autopsy. A team of cardiologists and neurologists made the final decision about cardiovascular and cerebrovascular accidents based on all the collected information for each patient. The loss to follow-up rate was 404 (6.4%), 249 (3.9%), and 191 (2.9%) for the second to third follow-ups, respectively. Despite this, a preliminary study of the individuals lost to follow-up, compared with a random sample of the remaining population, revealed no significant difference in basic characteristics (Supplementary Table S1). It’s worth noting that all participants provided written informed consent after receiving comprehensive explanations from qualified specialists.

### Dietary intakes assessment

In 2003, an expert dietitian obtained participants’ dietary intakes through face-to-face interviews using a 49-item qualitative Food Frequency Questionnaire (FFQ). The validity and reliability of the FFQ have been previously examined (23). For each food item, participants were asked, “During the past year, how often have you eaten these foods?” The questionnaire offered five predefined frequency categories: “never,” “rarely,” “daily,” “weekly,” and “monthly.” In our FFQ, no portion sizes were specified; however, our validation study demonstrated that discrepancies are mainly because of variations in the frequencies rather than the portion size (23). Hence, portion size data are rather unimportant, since most of the variation in food intake is justified by consumption frequency (23). To calculate dietary clusters, the 49 food items were categorized into 12 food groups based on nutrient similarities as follows: fruits (fresh fruit, fruit juice, and dried fruit), vegetables (fresh, cooked, and dried vegetables), dairy products (cheese, low and whole fat milk, and yogurt), legumes (lentils, peas, beans, etc.), nuts (almonds, hazelnuts, pistachios, and seeds), white meat (chicken, poultry, fresh fish), grains (bread, rice), red meat, processed meat (sausages and burgers), sweets (sweets, cake, biscuits, chocolate, and pizza), non-hydrogenated vegetable oils (solid oil), and hydrogenated vegetable oils (liquid oil, olive oil) (24). It is important to note that all grains were classified as unhealthy, primarily because they are largely refined. Furthermore, due to the lack of information on low- and high-fat dairy products, these were considered healthy foods in our study.

### Clinical and laboratory measurements

Participants were invited to their nearest health center, where a team of trained physicians and nurses conducted an extensive medical interview and physical examination. Subjects completed general questionnaires to gather information on demographics and risk factors for CVDs, including age, sex, family history of CVD, socioeconomic status, residency areas, anthropometric measures, physical activity, smoking status, hypertension, and dyslipidemia. Smoking status was categorized as a smoker, non-smoker, or ex-smoker (25). The Baecke questionnaire was used to assess physical activity, which was reported in equivalent metabolic minutes per week (METs-min/wk) (26). To estimate each person’s total METs-min/wk., we calculated the METs-min/wk. for each activity (days per week × MET equivalent of exercise minutes) and then summed up all METs-min/wk. values. Anthropometric and blood pressure measurements were performed following standard protocols (27, 28) using calibrated instruments. Abdominal obesity was determined by the waist-to-hip ratio (WHR), with cut-off values set at WHR ≥ 0.90 cm for men and WHR ≥ 0.85 cm for women. Obesity was determined by a body mass index (BMI) ≥ 30 kg. Hypertension was defined as individuals with systolic blood pressure (SBP) ≥ 140 mmHg, diastolic blood pressure (DBP) ≥ 90 mmHg, or those taking antihypertensive medication, as per WHO guidelines (29).

In this study, dyslipidemia was considered as an abnormal value of at least one of the lipid profile components, including total cholesterol (TC) ≥ 200 mg/dL, triglyceride (TG) ≥ 150 mg/dL, low-density lipoprotein cholesterol (LDL-C) ≥ 130 mg/dL, or high-density lipoprotein cholesterol (HDL-C) ≤ 40 mg/dL in men and HDL-C ≤ 50 mg/dL in women. Those taking lipid-lowering drugs were also classified as having dyslipidemia (30, 31).

### Statistical analysis

To cluster individuals based on their food intake and identify dietary patterns, we utilized the semi-parametric Bayesian approach of the Dirichlet Process (32). The Dirichlet Process is less susceptible to outliers and deviations from normality compared to the commonly used method of a mixture of Gaussian distributions. This was particularly useful when dealing with significant overlap between observations. The Dirichlet Process evaluates an unknown distribution G over all possible distributions, focused around a base distribution G0, with dispersion around this measure regulated by a dispersion parameter. Although G0 is typically assumed to be a Gaussian distribution, other distributions have been proposed [e.g., (20)]. The Dirichlet Process’ capacity to generate discrete distributions was employed by many researchers to cluster data with complex structures, as shown in previous studies (33–35). We implemented the Gaussian Dirichlet process mixture model (36, 37) to generate clusters. We considered the stick-breaking representation of the Dirichlet process for implementation in the OpenBUGs software (38). In our model-based clustering approach, each individual was assigned to the cluster with the highest probability density. Within a Bayesian framework, we estimated the distribution parameters of all clusters, calculated the probability density of individual observations in each cluster, and assigned each individual to the cluster with the highest density. We used the Markov chain Monte Carlo (MCMC) simulation method to obtain the Bayesian estimates of the parameters. After discarding 1,000 simulated values as burn-in for MCMC convergence, we averaged 20,000 MCMC samples for each parameter to obtain the Bayes estimates. The clustering approach was detailed previously (34, 39). We labeled the three main dietary clusters as healthy, unhealthy, and mixed.

In this study, we conducted all analyses on 5,396 individuals who completed all food-item questions on the FFQ. Continuous data were expressed as mean ± standard deviation (SD), and categorical data were presented as percentages. Differences between groups were evaluated by an independent samples t-test for continuous variables and a Chi-squared test for categorical variables. We used one-way analysis of variance (ANOVA) to compare continuous variables across three dietary clusters, and the Chi-square test for categorical variables.

We utilized Cox proportional hazards regression analysis to obtain the hazard ratio (HR) and 95% confidence intervals (CI) for the association between the derived dietary clusters and the incidence of CVDs. The first model was adjusted for age (continuous), gender, and socioeconomic status (low/moderate/high). The subsequent model was additionally adjusted for smoking (smoker/non-smoker/ex-smoker), physical activity (continuous), and general obesity (yes/no). Additional adjustments were made for residency areas (urban/rural), family history of CVDs (yes/no), abdominal obesity (yes/no), hypertension (yes/no), and dyslipidemia (yes/no).

## Results

Table 1 displays the demographic and clinical characteristics of the study participants, segmented by the incidence of cardiovascular events. We identified 401 males (15.3%) and 340 females (12.3%) with cardiovascular diseases (CVD). The prevalence of CVDs was significantly higher in males than females (*p* < 0.05). Factors such as mean age, anthropometric measures (WHR and BMI), and lipid profile components (LDL-C, TG, TC) were significantly higher in individuals with CVDs compared to those without CVDs (all p < 0.05). Notably, HDL levels were not significantly different between these two groups. Participants with CVDs exhibited lower physical activity levels, a higher proportion of urban residency, and generally had lower socioeconomic status.

**Table 1:** Demographic and clinical characteristics of the study population stratified by the development of cardiovascular events.

	Cardiovascular disease			
	Total (*n* = 5,396)	Yes (*n* = 741)	No (*n* = 4,655)	*p*-value
*Demographic variables*				
Age (year)	50.67 ± 11.61	57.31 ± 11.66	49.61 ± 11.25	0.001^a^
Sex				
Male	2,628 (48.7)	401 (54.1)	2,227 (47.8)	0.001^b^
Female	2,768 (51.3)	340 (45.9)	2,428 (52.2)	
Family history of CVD				
Yes	268 (4.6)	64 (8.7)	204 (4.3)	0.024^b^
No	5,128 (95.4)	677 (91.3)	4,451 (95.7)	
Socioeconomic status				
Low	5,353 (99.2)	733 (98.9)	4,622 (99.3)	0.032^b^
Moderate	27 (0.5)	2 (0.3)	23 (0.5)	
High	16 (0.3)	6 (0.8)	10 (0.2)	
Residency areas				
Urban	3,907 (72.4)	576 (77.7)	3,330 (71.6)	< 0.001^b^
Rural	1,489 (27.6)	165 (22.3)	1,325 (28.4)	
*Anthropometric measures*				
Waist-to-hip ratio	0.93 ± 0.8	0.95 ± 0.07	0.92 ± 0.08	< 0.001^a^
Body mass index (kg/m^2^)	26.79 ± 4.6	37.27 ± 4.84	26.69 ± 4.64	0.001^a^
*Lifestyle variables*				
Physical Activity (MET/min/wk)	873.44 ± 549.26	778.71 ± 563.54	888.72 ± 545.63	< 0.001^a^
Smoking status				
Smoker	842 (15.6)	129 (17.4)	712 (15.3)	0.006^b^
Ex-smoker	346 (6.4)	64 (8.6)	282 (6.1)	
Non-smoker	4,208 (78.0)	548 (74.0)	3,661 (78.6)	
*Co-complications*				
Hypertension				
Yes	755 (14.0)	205 (27.7)	550 (11.8)	< 0.001^b^
No	4,637 (86.0)	536 (72.3)	4,105 (88.2)	
HDL-C (mg/dL)	46.93 ± 10.45	47.05 ± 10.73	46.91 ± 10.41	0.901^a^
LDL-C (mg/dL)	128.43 ± 42.14	135.3 ± 45.34	127.3 ± 41.54	< 0.001^a^
TG (mg/dL)	193.42 ± 118.97	225.02 ± 51.64	210.3 ± 49.56	< 0.001^a^
Total cholesterol (mg/dL)	212.39 ± 50.1	222.13 ± 139.6	188.8 ± 114.6	< 0.001^a^

Data are presented as mean ± SD or number (%). *p* < 0.05 was considered statistically significant. CVD: Cardiovascular disease, MET: Metabolic Equivalents, HDL-C: High-density lipoprotein cholesterol, LDL-C: Low-density lipoprotein cholesterol, TG: Triglyceride.

a Calculated by an independent samples *t*-test.

b Calculated by the Chi-square test.

We identified three dietary clusters: healthy, unhealthy, and mixed. Table 2 demonstrates the dietary consumption of frequently consumed food items (consumed > times/portion/week), stratified by dietary clusters. The unhealthy cluster consumed more white meat, processed meat, sweets, non-hydrogenated vegetable oils, and hydrogenated vegetable oils. Conversely, the healthy cluster consumed more fruits, vegetables, dairy products, legumes, nuts, red meat, and grain.

**Table 2:** Dietary consumption of the most commonly consumed food items (>3 times/portion/week) stratified by the dietary clusters.

*Food groups*	Dietary clusters			
	Unhealthy (*n* = 97)	Mixed (*n* = 4,954)	Healthy (*n* = 345)	*p*-value^a^
Fruits (time/wk)	8.4 ± 5.5	7.4 ± 4.6	9.7 ± 5.7	< 0.001
Vegetables (time/wk)	6.6 ± 4.1	6.2 ± 3.8	7.4 ± 4.4	< 0.001
Dairy products (time/wk)	0.63 ± 1.9	0.65 ± 1.6	1.1 ± 2.3	< 0.001
Legumes (time/wk)	3.2 ± 2.9	3.03 ± 2.2	3.7 ± 2.7	< 0.001
Nuts (time/wk)	1.5 ± 2.2	0.6 ± 0.9	8.1 ± 3.5	< 0.001
White meat (time/wk)	2.7 ± 2.5	2.3 ± 2.2	2.5 ± 2.3	0.019
Red meat (time/wk)	3.7 ± 2.9	4.1 ± 2.7	4.8 ± 3.07	< 0.001
Processed meat (time/wk)	5.7 ± 2.2	0.3 ± 0.6	0.8 ± 1.1	< 0.001
Grain (time/wk)	23.9 ± 7.7	23.5 ± 6.02	24.3 ± 6.4	0.037
Sweets (time/wk)	5.7 ± 7.5	1.8 ± 3.1	4.7 ± 6.1	< 0.001
HVOs (time/wk)	9.3 ± 5.2	8.08 ± 4.8	8.5 ± 5.03	0.009
NHVOs (time/wk)	3.2 ± 4.6	2.4 ± 3.9	2.9 ± 4.7	0.021

Data are presented as mean ± SD. *p* < 0.05 was considered statistically significant. HVOs, Hydrogenated vegetable oils; NHVOs, Non-hydrogenated vegetable oils.

a Calculated by one-way analysis of variance (ANOVA).

Table 3 outlines the demographic and clinical characteristics of the study population, stratified by dietary clusters. Participants in the mixed cluster were typically older, more likely hypertensive, and had lower socioeconomic status and physical activity levels than those in healthy and unhealthy clusters. Moreover, the information across dietary clusters at different follow-up points was presented in Supplementary Table S2.

**Table 3:** Demographic and clinical characteristics of the study population stratified by dietary clusters.

	Dietary clusters			
	Unhealthy	Mixed	Healthy	*p-value*
*Demographic variables*				
Age (year)	45.23 ± 9.52	51.07 ± 11.68	46.47 ± 9.74	< 0.001^a^
Sex				
Male	46 (47.4)	2,421 (48.9)	160 (46.4)	0.640^b^
Female	51 (52.6)	2,533 (51.1)	185 (53.6)	
Family history of CVD				
Yes	5 (5.2)	223 (4.5)	20 (5.8)	0.520^b^
No	92 (94.8)	4,731 (95.5)	325 (94.2)	
Socioeconomic status				
Low	78 (97.5)	4,229 (99.3)	274 (98.6)	0.010^b^
Moderate	2 (2.5)	17 (0.4)	4 (1.4)	
High	0 (0.0)	13 (0.3)	0 (0.0)	
Residency areas				
Urban	79 (81.4)	3,589 (72.4)	239 (69.3)	0.059^b^
Rural	18 (18.6)	1,365 (27.6)	106 (30.7)	
*Anthropometric measures*				
Waist-to-hip ratio	0.925 ± 0.084	0.93 ± 0.080	0.921 ± 0.089	0.052^a^
Body mass index (kg/m^2^)	27.65 ± 4.5	26.76 ± 4.7	26.9 ± 4.3	0.160^a^
*Lifestyle variables*				
Physical Activity (MET/min/wk)	960.01 ± 503.64	867.91 ± 548.94	928.50 ± 562.46	0.041^a^
Smoking status				
Smoker	21 (21.6)	760 (15.3)	60 (17.4)	0.350^b^
Ex-smoker	7 (7.2)	320 (6.5)	19 (5.5)	
Non-smoker	69 (71.2)	3,874 (78.2)	266 (77.1)	
*Co-complications*				
Hypertension				
Yes	8 (8.2)	714 (14.4)	34 (9.9)	0.010^b^
No	89 (91.8)	4,240 (85.6)	311 (90.1)	
HDL-C (mg/dL)	45.88 ± 10.28	47.00 ± 10.43	46.21 ± 10.85	0.250^a^
LDL-C (mg/dL)	123.92 ± 44.85	128.6 ± 41.93	127.36 ± 44.45	0.520^a^
TG (mg/dL)	208 ± 50.05	212.58 ± 49.93	210.9 ± 52.5	0.570^a^
Total cholesterol (mg/dL)	195.42 ± 104.69	192.53 ± 114.59	205.63 ± 171.88	0.140^a^

Data are presented as mean ± SD or number (%). *p* < 0.05 was considered statistically significant. CVD, Cardiovascular disease; MET, Metabolic Equivalents; HDL-C, High-density lipoprotein cholesterol; LDL-C, Low-density lipoprotein cholesterol; TG, Triglyceride.

a Calculated by one-way analysis of variance (ANOVA).

b Calculated by the Chi-square test.

The study sample comprised 5,396 subjects, including 741 with CVDs. Of the 97 individuals in the unhealthy dietary cluster, 13 (13.4%) had CVDs, while 84 (86.6%) were censored. Among 4,954 participants in the mixed dietary cluster, 689 (13.9%) had CVDs, with 4,265 (86.1%) censored. In the healthy dietary cluster, 39 out of 345 participants (11.3%) had CVDs, and 306 (88.7%) were censored. Loss of follow-up was due to various factors such as changes in phone numbers, addresses, and geographical challenges. These losses were considered random and not biased. Detailed reasons for participant drop-outs are presented elsewhere (40).

**Figure 1:**
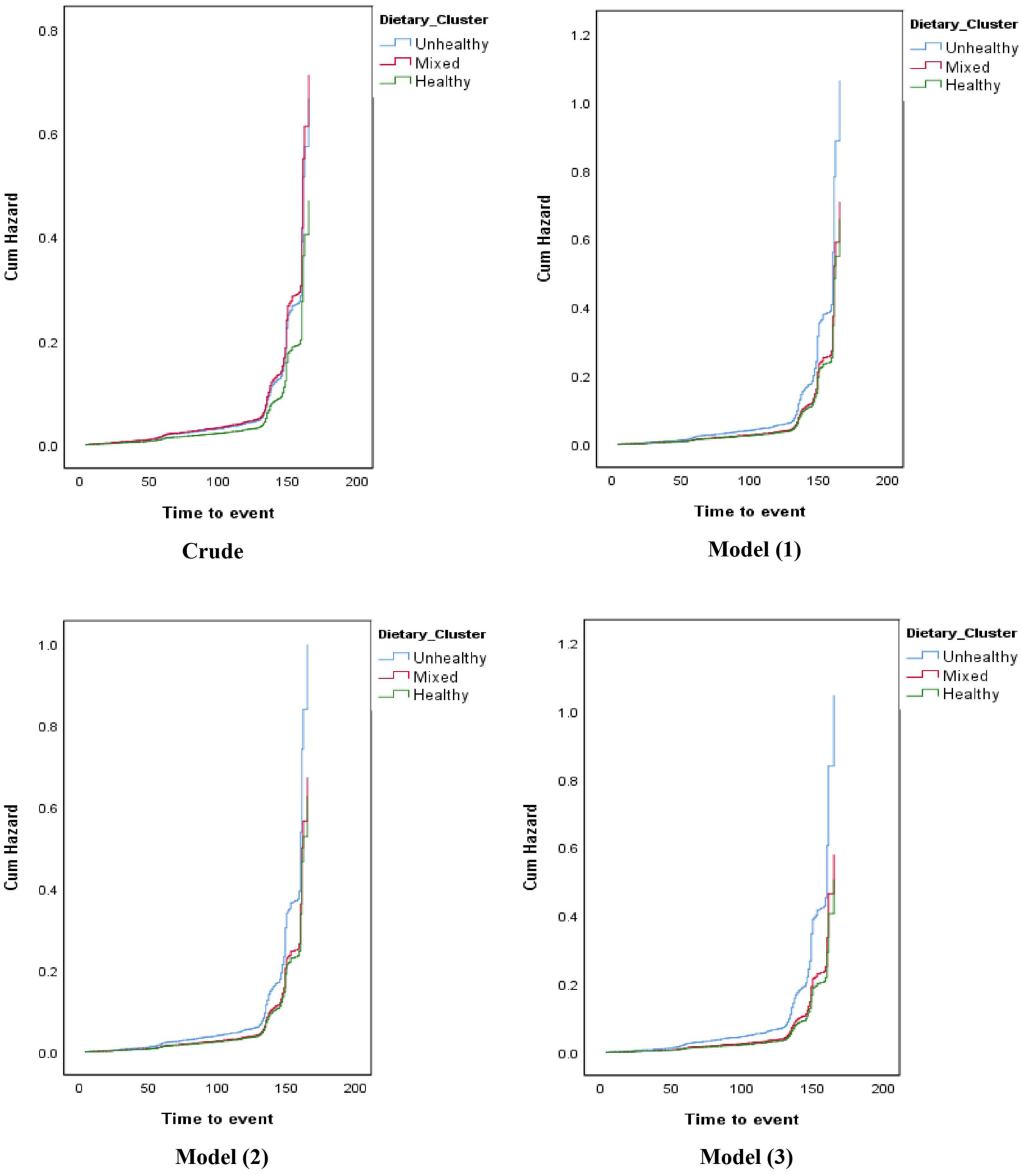
Hazard ratio (HR) for the association between dietary clusters and the risk of cardiovascular events.

Table 4 shows the hazard ratio (HR) and corresponding 95% confidence intervals (CI) for the association between dietary clusters and CVD risk. In the crude model, participants in the mixed cluster had a higher CVD risk than those in the healthy cluster (HR: 1.515; 95%CI: 1.097, 2.092). However, after adjusting for age, gender, socioeconomic status, smoking, physical activity, general obesity, residency areas, family history of CVDs, abdominal obesity, hypertension, and dyslipidemia, these findings were attenuated (HR: 1.145; 95%CI: 0.769, 1.706). The crude model did not show a significant association between the unhealthy dietary cluster and cardiovascular event risk compared to the healthy cluster (HR: 1.418; 95%CI: 0.757, 2.658). Nevertheless, the fully-adjusted model indicated that participants in the unhealthy cluster had a higher CVD risk than those in the healthy cluster (HR: 2.059; 95%CI: 1.013, 4.184) (Figure 1).

**Table 4:** Hazard ratio (HR) and 95% confidence interval (CI) for the association between dietary clusters and the risk of cardiovascular disease.

Dietary clusters	*p*-value	HR (95% CI)
Crude		
Healthy	–	Ref
Unhealthy	0.276	1.418 (0.757, 2.658)
Mixed	0.012	1.515 (1.097, 2.092)
Model-1		
Healthy	–	Ref
Unhealthy	0.173	1.615 (0.811, 3.216)
Mixed	0.691	1.077 (0.748, 1.550)
Model-2		
Healthy	–	Ref
Unhealthy	0.187	1.591 (0.798, 3.171)
Mixed	0.708	1.072 (0.744, 1.544)
Model-3		
Healthy	–	Ref
Unhealthy	0.046	2.059 (1.013–4.184)
Mixed	0.506	1.145 (0.769–1.706)

Data are presented as HR (95% confidence interval) and obtained from Cox proportional hazard regression analysis. *p* < 0.05 was considered statistically significant. Crude: Unadjusted. Model 1: Adjusted for age, sex, and socioeconomic status. Model 2: Model 1+ smoking status, physical activity, general obesity. Model 3: Model 2 + residency areas, family history of cardiovascular disease, abdominal obesity, hypertension, and dyslipidemia.

## Discussion

Through the use of Bayesian cluster analysis, we identified three primary dietary patterns among the Iranian adult population: ‘unhealthy’, ‘mixed’, and ‘healthy’. The analysis revealed that individuals within the unhealthy cluster had nearly twice the risk for cardiovascular diseases (CVDs), independent of potential confounders. Furthermore, the association between mixed clusters and a higher risk for CVDs was found to be influenced by variables such as age, gender, socioeconomic status, smoking habits, level of physical activity, general obesity, residency areas, family history of CVDs, abdominal obesity, hypertension, and dyslipidemia.

Our findings suggest that adopting a healthy dietary pattern, similar to the one identified in this study, could significantly reduce the risk of CVDs. This conclusion aligns with the results of other research, showing a positive association between the consumption of vegetables, fruits, and dairy products and improvements in variables such as lipid profile, adiposity measures, diastolic blood pressure (DBP), and fasting glucose concentration (41).

Moreover, our findings align with studies highlighting the benefits of a dietary pattern characterized by high vegetable consumption, often referred to as ‘healthy’ or ‘cautious’, in improving cardiovascular risk factors (42, 43). A separate study conducted among Brazilian adults found that adherence to a healthy dietary pattern led to favorable outcomes related to obesity, including lower waist circumference (WC), Body Mass Index (BMI), and Waist-to-Hip Ratio (WHR), along with lower systolic blood pressure (SBP) and improved lipid profile (44).

Moreover, adherence to a diet rich in fruits and vegetables has been linked to a higher intake of dietary fiber, folate, and potassium (45–47). Fruits and vegetables are known to contain biologically active compounds such as flavonoids and antioxidants, which may have beneficial effects against CVDs (45–48). While the exact cardioprotective properties of these substances are not fully understood, their consumption has been linked to a reduced risk of CVDs (46–49).

Various studies have further demonstrated the beneficial effects of fruit and vegetable consumption in preventing CVDs (50, 51). Data from a cohort study conducted in Sudan has shown that lifestyle modifications, including a healthy diet, could potentially prevent up to 80% of CVDs (52, 53). This underscores the importance of population-based strategies focusing on lifestyle modifications, including nutrition, in CVD prevention (54, 55).

In recent years, numerous studies have been conducted to investigate the effects of different food groups on CVD prevention and related mechanisms. These studies suggest that certain food groups, such as fruits, vegetables, legumes, nuts, grains, low-fat dairy products, and fish, confer beneficial effects in preventing CVDs (29). Recent research from the Swedish Mammography Cohort has demonstrated that adherence to a healthy dietary pattern can significantly lower the risk of myocardial infarction (MI) (56).

A longitudinal analysis conducted among Finnish adults underscored that adherence to a healthy eating pattern, characterized by high consumption of vegetables, legumes, nuts, tea, rye, and dairy products, was associated with reduced cardiovascular risk factors (57). In a cross-sectional study by Amini et al., the relationship between dietary patterns and visceral adiposity, lipid accumulation product (LAP), and triglyceride-glucose (TyG) index was examined among adults aged 18–45 years living in Tehran (58). The healthy dietary pattern in this study included vegetables, fruits and fruit juices, legumes, poultry, nuts, fish, eggs, low-fat dairy products, olives, and olive oil. The results revealed no significant associations between the healthy dietary pattern, TyG index, and visceral adiposity index, after adjusting for potential confounders. However, stronger adherence to the healthy dietary pattern was associated with a decrease in LAP (58).

In a distinct population-based cross-sectional study, Sauvageot et al. used stability-based validation of dietary patterns acquired through cluster analysis to investigate the association between these patterns and cardiovascular risk factors (59). This study suggested that a healthy dietary pattern was less associated with cardiovascular risk factors compared to an unhealthy one. The unhealthy dietary pattern was linked to higher systolic blood pressure (SBP) and diastolic blood pressure (DBP), and a significant association with fasting plasma glucose (FPG), but not with hemoglobin A1C (HbA1C) levels (59).

In a meta-analysis of observational studies, there was no observed association between saturated fatty acid (SFA) intake and the risk of CVDs or type 2 diabetes, or with all-cause and CVD mortality. However, the 2015 Dietary Guidelines Advisory Committee’s scientific report proposed that replacing 1% of energy intake from SFA with polyunsaturated fatty acids (PUFA) may reduce the incidence of coronary heart disease by 2–3% (60, 61). Conversely, trans fatty acids, which are primarily found in products like cakes, cookies, and salad dressings, and mainly derived from the hydrogenation of industrial vegetable oils, have been associated with an increased risk of CVDs in numerous clinical studies (62–66). Trans fatty acid intake has been shown to elevate LDL-C levels, decrease HDL levels, and increase inflammation and endothelial dysfunction (67–69).

Consumption of an unhealthy diet has been linked with a higher intake of processed meat, which is known to contain 400% more sodium than raw meat (70). High sodium intake is a recognized risk factor for CVDs due to its potential to elevate blood pressure (71). Hence, reducing processed meat consumption could contribute to lowering the incidence of CVDs. A study by Rouhani et al. (72) explored the correlation between fast-food consumption and obesity among adolescent girls in Isfahan province and found that higher consumption of fast food was linked to lower diet quality, leading to increased cardiovascular risk factors such as dyslipidemia. Fast foods are typically energy-dense and are rich in saturated and trans fats. Recent research revealed that trans fats account for 24 and 31% of total fats in typical sausages and burgers in Iran, respectively (73). Saturated and trans fats have been demonstrated to negatively impact lipid profiles, metabolic syndromes, systemic inflammation, and endothelial function (74).

Research indicates compelling evidence supporting the assertion that red meat, a significant source of protein and fat, may not be optimal for dietary health due to predicted increases in total cholesterol (TC) and LDL-C (75). As a result, dietary health recommendations often limit red meat consumption or suggest replacing it with leaner options such as white meat (75). Several systematic reviews and meta-analyses have revealed an inverse relationship between red meat consumption and overall health (76). Additionally, red meat consumption has been linked to non-communicable diseases (NCDs) (75). For instance, World Cancer Research has reported strong evidence connecting the consumption of red and processed meat to an increased risk of colorectal cancer (77). Studies have also shown associations between red meat consumption and type 2 diabetes (78) and cardiovascular diseases (CVDs) such as coronary artery disease, stroke, and heart failure (70, 75, 79).

Our research further indicated that most individuals within unhealthy dietary clusters have poor social and economic statuses, consistent with previous study findings (80–82). Research has demonstrated that individuals with higher socioeconomic status tend to follow healthier food shopping patterns than those with lower socioeconomic status. For instance, a 2010 survey of British families revealed that lower socioeconomic status groups purchased more energy from less healthy foods and beverages, while those of higher status prioritized healthier food choices (82). Furthermore, international research in Latin America showed that participants with low socioeconomic status consumed less fruit, vegetables, whole grains, fiber, fish, and shellfish, but consumed more legumes than those with higher status (83). Lower adherence to the Mediterranean diet has also been observed in groups with lower socioeconomic status (84). The higher cost of healthier diets may pose a barrier for low-income individuals to adopt healthy eating habits, as diets rich in quality and nutritious foods, such as lean meats, fish, vegetables, and fruits, are often more expensive than diets high in added sugars, fats, and refined grains (81). For example, an American study found that a healthy food basket could cost $14 to $32 more than a standard food basket due to the higher costs of whole grains, lean meats, and skinless chicken, which could constitute over 35 to 40 percent of the food budget of low-income consumers (85). This underscores the challenges faced by individuals with lower socioeconomic status in making healthy food choices due to financial constraints. These findings suggest that socioeconomic status significantly shapes dietary patterns, and addressing economic and social factors is vital in promoting healthy eating habits for cardiovascular disease prevention and management (80–85).

The results of our study suggest that individuals with unhealthy dietary patterns tend to be younger, aligning with findings from previous research (86–88). For instance, a study conducted in Spain found that younger people tend to follow a Western diet, characterized by high consumption of red and processed meat, refined grains, fried food, and high-fat foods. This dietary pattern is considered unhealthy in contrast to the Mediterranean diet, which is regarded as a healthy dietary pattern and includes more fruits, seafood, legumes, and nuts (87). Another study also demonstrated an inverse relationship between age and healthy eating patterns, with younger individuals more inclined to follow unhealthy eating patterns than older individuals (88). This may be attributed, in part, to the higher consumption of unhealthy foods, such as hamburgers and pizza, among young people (87). These findings suggest that age plays a role in shaping dietary patterns, with younger individuals being more susceptible to unhealthy eating patterns. Understanding these age-related differences in dietary choices can inform targeted interventions to promote healthy eating habits among younger populations for cardiovascular disease prevention.

The current study on dietary patterns in developing countries is scarce and often limited to cross-sectional studies. However, the present study, based on a population-based sample, enhances the external validity of the findings. Additionally, the study utilizes the Bayesian clustering method in a cohort study, which represents a novel approach.

However, some limitations should be considered when interpreting the results. The choice of variables used for clustering can affect the results, and different variables may yield different clustering outcomes. Nonetheless, clustering can be a valuable tool for identifying patterns in data, including dietary patterns and providing insights for further analysis and interpretation. In this regard, it is important to note that the nutritional performance of individuals in this study was determined using a standard food frequency questionnaire, which may have limitations such as a lack of access to detailed food intake information. This limitation could have affected the categorization of food items into food groups. For example, grains should ideally be divided into whole and refined grains, but in this study, all available grains were classified as unhealthy due to the prevalence of refined grains in Iran. Another limitation is that the questionnaire did not differentiate between low-fat and high-fat dairy products, resulting in all dairy products being categorized as healthy. Although our FFQ was validated, it did not provide us with data on portion sizes. Therefore, we did not have any data about total energy intake in this study.

The foundation of combating non-communicable diseases, including cardiovascular diseases (CVDs), lies in empowering individuals and policymakers through education and implementing supportive laws and regulations to create an environment conducive to healthy behaviors and lifestyles. By implementing the right societal interventions, we can eliminate or reduce many risk factors for CVDs. Community-based interventions that target risk factors such as smoking, unhealthy diets (high in fat but lacking in fruits and vegetables), physical inactivity, and other environmental, social, and behavioral variables are suggested for prevention.

In conclusion, dietary clusters within the Iranian population can be associated with the risk of CVDs. An essential aspect of the “health transition” is the emergence of CVD patterns associated with changes in dietary patterns, which should alert policymakers. Further prospective investigations are required to confirm such associations.

## Data availability statement

The raw data supporting the conclusions of this article will be made available by the authors, without undue reservation.

## Ethics statement

The studies involving human participants were reviewed and approved by The current study was conducted in accordance with the ethical guidelines of the 1975 Declaration of Helsinki and the protocol was approved by the Ethical Committee of Isfahan Cardiovascular Research Center. The patients/participants provided their written informed consent to participate in this study.

## Author contributions

NS, MM, NM, and RR: conception and design. RR, NM, and KS: acquisition of data. MM, RR, KS, and ER: analysis and interpretation of data. RR, KS, MM, and AA: drafting the manuscript. RR, KS, ER, AA, FN, NM, MM, and NS: revising it for intellectual content. All authors contributed to the article and approved the submitted version.

## Acknowledgments

The authors thank all patients who kindly contributed to the study.

## Conflict of interest

The authors declare that the research was conducted in the absence of any commercial or financial relationships that could be construed as a potential conflict of interest.

## Publisher’s note

All claims expressed in this article are solely those of the authors and do not necessarily represent those of their affiliated organizations, or those of the publisher, the editors and the reviewers. Any product that may be evaluated in this article, or claim that may be made by its manufacturer, is not guaranteed or endorsed by the publisher.
