# Supplementary material for: Population food intake clusters and cardiovascular disease incidence: a Bayesian quantifying of a prospective population-based cohort study in a low and middle-income country
Source: Front Nutr. 2023 Jul 13;10:1150481. doi: 10.3389/fnut.2023.1150481 (PMC10374205; doi:10.3389/fnut.2023.1150481)
Supplement: Supplementary file 1 [file Data_Sheet_1.docx]

**Supplementary Table 1**. Comparison between subjects remaining to the end of the study and those who lost to follow up

| Basic characteristics | Total subjects (N=5396) | | *p-value* |
| --- | --- | --- | --- |
|  | Subjects who remained in the study (n=4,552) | Subjects who were lost (n=844) |  |
| Age (year) | 53.55 ± 10.57 | 53.75 ± 12.14 | 0.388 |
| Sex |  |  |  |
| Male | 2199 (48.3) | 413 (48.9) | 0.516 |
| Female | 2353 (51.7) | 431 (51.1) |  |
| Family history of CVD |  |  |  |
| Yes | 4306 (94.6) | 801 (94.9) | 0.547 |
| No | 246 (5.4) | 43 (5.1) |  |
| Socioeconomic status |  |  |  |
| Low | 3450 (75.8) | 738 (87.5) | 0.088 |
| Moderate | 778 (17.1) | 73 (8.6) |  |
| High | 324 (7.1) | 33 (3.9) |  |
| Residency areas |  |  |  |
| Urban | 3824 (84.0) | 582 (69.0) | 0.064 |
| Rural | 644 (16.0) | 262 (31.0) |  |
| Anthropometric measures | | | |
| Waist-to-hip ratio | 0.94 ± 0.7 | 0.93 ± 0.07 | 0.083 |
| Body mass index (kg/m^2^) | 27.77 ± 4.65 | 26.74 ± 4.63 | 0.076 |
| Lifestyle variables | | | |
| Physical Activity (MET/min/wk) | 900.76 ± 562.16 | 811.14 ± 598.37 | 0.077 |
| Smoking status |  |  |  |
| Smoker | 1,493 (36.5) | 153 (18.1) | 0.065 |
| Ex-smoker | 256 (6.3) | 47 (5.6) |  |
| Non-smoker | 2,346 (57.3) | 644 (76.3) |  |
| Co-complications | | | |
| Hypertension |  |  |  |
| Yes | 588 (14.3) | 102 (14.2) | 0.793 |
| No | 3,510 (85.7) | 724 (85.8) |  |
| HDL-C (mg/dL) | 47.32 ± 36.91 | 46.72 ± 25.15 | 0.316 |
| LDL-C (mg/dL) | 123.3 ± 34.60 | 124.8 ± 40.13 | 0.056 |
| TG (mg/dL) | 175.9 ± 109.13 | 185.09 ±115.96 | 0.089 |
| Total cholesterol (mg/dL) | 209.41 ± 45.13 | 211.54 ± 49.53 | 0.077 |

Data are presented as mean ± SD or number (%)

P<0.05 was considered statistically significant.

^1^ Calculated by an independent samples t-test

^2^ Calculated by the Chi-square test

CVD: Cardiovascular disease, MET: Metabolic Equivalents, HDL-C: High-density lipoprotein cholesterol, LDL-C: Low-density lipoprotein cholesterol, TG: Triglyceride

**Supplementary Table 2**. Ratios of average consumption of the food items in 2001, 2007, and 2013.

| **Food items ratio*** | **Clusters** | **2003** | **2007** | **2013** |
| --- | --- | --- | --- | --- |
| Nuts | Unhealthy | 0.15 | 0.32 | 0.24 |
|  | Mixed | 0.06 | 0.30 | 0.32 |
|  | Healthy | 0.79 | 0.38 | 0.45 |
| Fruits | Unhealthy | 0.33 | 0.32 | 0.31 |
|  | Mixed | 0.29 | 0.34 | 0.34 |
|  | Healthy | 0.38 | 0.34 | 0.35 |
| Vegetables | Unhealthy | 0.33 | 0.36 | 0.36 |
|  | Mixed | 0.31 | 0.32 | 0.32 |
|  | Healthy | 0.37 | 0.32 | 0.32 |
| Dairy products | Unhealthy | 0.26 | 0.35 | 0.17 |
|  | Mixed | 0.27 | 0.33 | 0.44 |
|  | Healthy | 0.46 | 0.33 | 0.38 |
| Legumes | Unhealthy | 0.32 | 0.33 | 0.34 |
|  | Mixed | 0.30 | 0.33 | 0.32 |
|  | Healthy | 0.37 | 0.34 | 0.34 |
| White meat | Unhealthy | 0.36 | 0.38 | 0.29 |
|  | Mixed | 0.30 | 0.31 | 0.36 |
|  | Healthy | 0.34 | 0.32 | 0.36 |
| Grain | Unhealthy | 0.33 | 0.35 | 0.30 |
|  | Mixed | 0.33 | 0.32 | 0.33 |
|  | Healthy | 0.34 | 0.33 | 0.37 |
| Red meat | Unhealthy | 0.30 | 0.32 | 0.41 |
|  | Mixed | 0.33 | 0.34 | 0.32 |
|  | Healthy | 0.38 | 0.34 | 0.27 |
| Processed meat | Unhealthy | 0.82 | 0.49 | 0.69 |
|  | Mixed | 0.06 | 0.20 | 0.13 |
|  | Healthy | 0.12 | 0.31 | 0.17 |
| Sweets | Unhealthy | 0.47 | 0.36 | 0.42 |
|  | Mixed | 0.15 | 0.28 | 0.25 |
|  | Healthy | 0.38 | 0.35 | 0.33 |
| HVOs | Unhealthy | 0.36 | 0.34 | 0.37 |
|  | Mixed | 0.31 | 0.33 | 0.28 |
|  | Healthy | 0.33 | 0.33 | 0.35 |
| NHVOs | Unhealthy | 0.38 | 0.34 | 0.40 |
|  | Mixed | 0.29 | 0.32 | 0.31 |
|  | Healthy | 0.34 | 0.34 | 0.29 |

*The ratios were obtained by dividing the mean value of each food item within each cluster and each year by the sum of the mean values of that food item across all three clusters in that year.

HVOs: Hydrogenated vegetable oils, NHVOs: Non-hydrogenated vegetable oils.
